# Application of polymer integration technique for enhancing polyacrylamide (PAM) performance in high temperature and high salinity reservoirs

**DOI:** 10.1016/j.heliyon.2019.e02113

**Published:** 2019-07-23

**Authors:** Kingsley Godwin Uranta, Sina Rezaei Gomari, Paul Russell, Faik Hamad

**Affiliations:** School of Science, Engineering and Design, Teesside University, United Kingdom

**Keywords:** Chemical engineering, Polyacrylamide (PAM), Polyvinylpyrrolidone (PVP), 2-Acrylamido-2-MethylpropaneSulfonic acid (AMPS), High salinity, High temperature, Polymer integration

## Abstract

Polyacrylamides (PAM) are widely used as water-soluble polymers producing gel in oil reservoirs to assist in oil extraction from reservoirs with high levels of heterogeneity. These gels are susceptible to degradation due to hydrolysis in harsh reservoir conditions such as elevated temperature and salinity. This study uses a polymer integration technique in attempting to optimize the performance of PAM in the enhanced oil recovery process for reservoirs with high temperature and salinity. The results show that, at high temperature, hydrolysis is suppressed and gel stability is maintained via the addition of Polyvinylpyrrolidone (PVP) to PAM solutions.

The optimum composition was identified as being 20/80 wt% PAM: PVP for oilfield operations at 90 °C and a moderate salinity of 43,280 ppm. The degree of hydrolysis at 30 days was suppressed from 75% to 29.9%, with associated increases in viscosity from 11 to 38.2 mPa.s and from 18 to 44.3 mPa.s corresponding to rotational speeds of 30 and 10 rpm respectively.

The issue of high salinity was considered by increasing the salinity of the optimised PAM: PVP mixture to 200,000 ppm. Under these conditions the degree of hydrolysis of the optimised solution increased from 29.9 to 46.9% and viscosity decreased from 38.2 to 28.6 and from 44.3 to 40.4 mPa.s for rotational speeds of 30 and 10 rpm respectively. 2-Acrylamido-2-MethylpropaneSulfonic acid (AMPS) was added to the mix to try to improve temperature stability. It was observed that, with an optimum composition of 18/72/10 wt% PAM:PVP:AMPS, the degree of hydrolysis decreased to 22% with viscosity levels of 30.6 and 22.8 mPa.s corresponding to rotational speeds of 10 and 30 rpm respectively.

## 1 Introduction

Incremental oil recovery in waterflood projects may be obtained by adding water soluble polymers of high molecular weight to the injection water so as to improve the mobility ratio and as well improving the volumetric sweep efficiency of the displacement process for both sandstone and carbonate reservoirs [1]. This can be achieved by increasing the viscosity of the injected water in order to reduce the mobility of the water phase, and thereby sweeping the oil bank to produce significant amounts of oil from reservoirs [2]. High molecular weight water-soluble compounds such as polyacrylamide (PAM) or hydrolysed polyacrylamide (HPAM) are the most commonly utilised polymers in enhanced oil recovery (EOR) operations in oilfields [2, 3]. These polymers are prone to degradation due to hydrolysis in harsh reservoir conditions such as elevated temperature and salinity [4]. In fact the amide group hydrolyses at elevated temperature to form carboxylate groups which in turn interact with bivalent salts (CaCl_2_, MgCl_2_) leading to sharp reductions in polymer solubility and solution viscosity [5, 6].

Various researchers worldwide have reported on the extent of degree of hydrolysis, and recommended a temperature range of 70–82 °C as the maximum safe temperature for PAM operations in seawater and other brines during polymer flooding [2, 5, 6, 7, 8, 9, 10]. Recently, Uranta et al.‘s [11] correlation analysis determined the safe maximum temperature point (SMTP) for polyacrylamide (PAM) in saline solutions, and the results imply that the above-mentioned temperature range is not suitable for every saline or brine solution. Apparently the SMTP for PAM could change depending on the salinity of the formation water or reservoir salt solution. Meanwhile Zaitoun and Potie's [8] analysis proved that precipitation or degradation could occur when the degree of hydrolysis exceeds 35% for a temperature of 30 °C or 33% for 80 °C.

Uranta et al. [12] reported that the degree of hydrolysis increases as temperature increases, with levels of hydrolysis at 53%, 65% and 75% observed for temperatures of 50, 70 and 90 °C respectively for the saline solution, along with corresponding drastic decreases in the viscosity of the solution.

One of the criteria for improving the performance of water-soluble polymers during EOR polymer flooding applications is to maintain the degree of hydrolysis below 33%. Above this level, degradation or precipitation may occur [3, 8]. This is due to the consequences of interaction between the hydrolysed amide group and the salinity of the formation water containing divalent cations. This chemical transformation in PAM could cause significant losses in solution viscosity, and separation could eventually occur in extreme conditions with high degrees of hydrolysis or concentrations of formation salinity [6]. The primary mechanism behind PAM degradation has been found to be the hydrolysis of amide groups, which in turn is a function of temperature [1, 9].

This work aims to address the instability or degradation of PAM at high temperature in moderate and high brines salinity which apparently are found to be a consequences of hydrolysis of PAM amide functional groups. A synthetic approach called the polymer integrated technique (PIT) was adopted in order to improve the stability of PAM against high temperature and salinity conditions. This approach is defined as a process of combining high molecular weight polymer with another polymer (or polymers) to meet the stability limitations or degradation of polymer at reservoir conditions. Two specific polymers were selected for integration with PAM: Polyvinylpyrrolidone (PVP) to protect acrylamide against thermal hydrolysis [13, 14], and 2-Acrylamido-2-methypropane sulfonate (AMPS) to enhance PAM tolerance to high formation water salinity and divalent ions [1, 15, 16, 17, 18, 19, 20].

## 2 Materials and methods

The approach used focuses on improving PAM performance in handling challenging reservoir conditions of high temperatures and high salinity and is achieved experimentally in three steps. In the first step, PVP was integrated with PAM at different concentrations in a moderate salinity of 43,280 ppm and at a temperature of 90 °C. The level of stability of PAM for each concentration was then determined. The optimal weight ratio of the polymers was selected based on a balance between acceptable solution viscosity and the lowest possible degree of hydrolysis. In the second step, the optimised mixture of PAM and PVP at a temperature of 90 °C was exposed to higher salinity formation brine with a TDS of 200,000 ppm, and the effects on brine stability and degree of hydrolysis were observed. Finally, 2-acrylamido-2-methylpropanesulphonic acid (AMPS) was added to the optimised mixture of PAM and PVP. Different weight ratios of PAM: PVP: AMPS were screened, and the optimal composition was found for high temperature (90 °C) and high salinity up to 200,000 ppm.

### 2.1 Preparation of integrated polymers

**Fig. 1:**
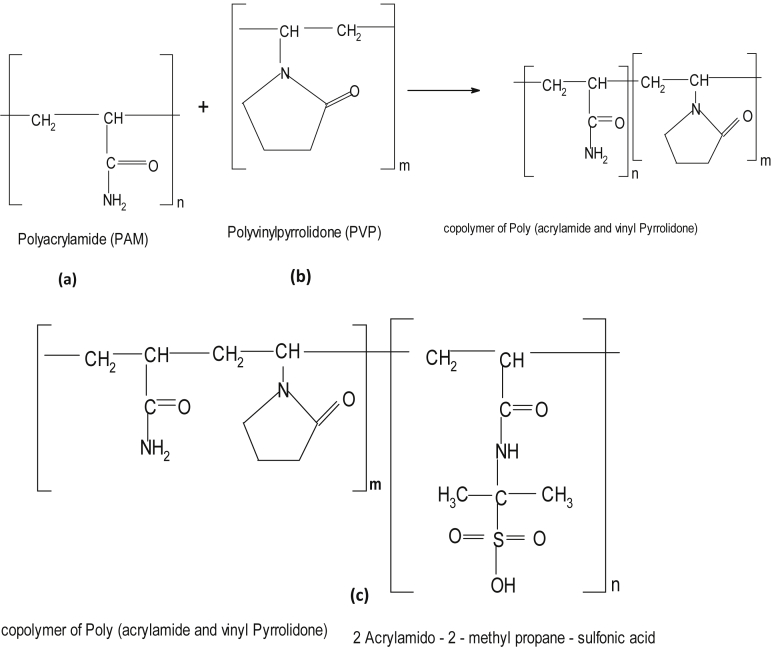
Molecular structure of (a) PAM, (b) PVP and (c) AMPS.

All polymers were purchased from Sigma-Aldrich. The structures of the selected polymers/co-polymers are presented in Fig. 1. The PAM solution was prepared using an overall composition of 1% (w/v) 10g concentration in 1000 ml beakers containing formation water with moderate salinity of 43,280 ppm and mixed with electric stirrer for 3 h. The prepared PAM solutions were then first mixed with PVP to enhance its resistance at a temperature of 90 °C, and later the optimal solutions of PAM and PVP were mixed with AMPS to enhance stability in highly saline water at 200,000 ppm.

Table 1 presents the various weight ratios of polymer mixtures dissolved in synthetic brine. In the various weight ratios shown in Table 1, the symbol A represents PAM:PVP mixtures and B represents PAM:PVP:AMPS mixtures. Table 2 shows the salinity of the synthesized formation water with total dissolved salts (TDS) of 43,280 and 200,000 ppm representing moderate and extreme salt concentrations respectively. Due to the presence of oxygen in the PIT mix solution, 1% (w/v) of Sodium thiosulphate (Na_2_S_2_O_3_) was added to the polymer solution as an oxygen scavenger.

**Table 1:** Various weight ratio of polymer mixtures dissolved in synthetic brine (symbol A representing the PAM: PVP mixtures and symbol B representing the PAM: PVP: AMPS mixtures).

Sample No:	Weight ratio composition (wt %)	Sample No:	Weight ratio composition (wt %)
1	A100/0	1	B20/80/0
2	A90/10	2	B19/76/5
3	A80/20	3	B18/72/10
4	A70/30	4	B10/40/50
5	A60/40	5	B2/8/90
6	A50/50	6	B0/0/100
7	A40/60		
8	A30/70		
9	A20/80		
10	A15/85		
11	A10/90		
12	A5/95		
13	A0/100

**Table 2:** Composition of synthetic brine.

Ion	Moderate salt concentration (ppm)	Extreme salt concentration (ppm)
NaCl	34700	170,000
CaCl_2_. 6H_2_O	4900	15000
MgCl_2_.6H_2_O	2700	10000
KCl	400	2500
NaHCO_3_	400	1500
SrCl_2_.6H_2_O	120	600
BaCl_2_.6H_2_O	60	400
Total Dissolved Salts (TDS)	43280	200,000

### 2.2 Rheology stability of the integrated polymers

Having prepared the integrated polymers, their rheological stability was tested by measuring the initial viscosity of the fresh samples using a Cole Parmer rotational viscometer at rotational speeds of 10 and 30 rpm with corresponding shear rates of 17 and 51 s^-1^ respectively. All weight ratios of polymer solution were aged in an oven at 90 °C and sampled at time intervals of 0, 1, 2, 4, 10, 20 and 30 days to allow measurements of the degree of hydrolysis and viscosity. The viscosity test for each weight ratio was conducted three times and the mean of the three viscosity measurements presented as the final viscosity.

### 2.3 Degree of hydrolysis of integrated polymers

The level of hydrolysis of amide groups has been found to be the primary mechanism in the degradation of water-soluble polymers, and it is worth emphasizing that degree of hydrolysis has been found to depend on temperature [9]. The process of measuring the degree of hydrolysis of aged samples at the elevated temperature of 90 °C involved the utilisation of the proton nuclear magnetic resonance (^1^H NMR) technique. The technique was utilised to measure the polymer samples collected at time zero to set the initial degree of hydrolysis and to speed up the analysis, Fourier transform infrared (FT-IR) technique was utilised to measure the change in amide group absorbance after each samples ageing and it was conducted three times and the mean of amide group absorbance measurements presented as the final percentage change in amide absorbance after each samples ageing.

The absolute degree of hydrolysis from the sample collected at zero hours was measured using ^1^H NMR, where 20 mg of PAM solution was dissolved in 1 ml of deuterium oxide (D_2_O) in a small vial which was then placed on a hot block at a temperature of 90 °C for over 3 h. The mixed solution was then transferred from the vial to an NMR tube. The NMR tube was inserted into a Bruker Advance III 400 MHz triple-NMR spectrometer with fast magnetic angle spinning (MAS) at 65 kHz and a rotor diameter between 1.3 and 4 mm. The results from the NMR technique for the initial degree of hydrolysis were analysed using Bruker Topspin 3.5 software via identification of the peak area assigned to the equivalent hydrogen (H) atom of the methine (=CH−) group bonded to the amide group (CONH_2_) and that of the methylene (CH_2_) group of the water soluble polymer, which act as prominent functional groups.

Fourier transform infrared (FT-IR) analysis was used to measure the change in degree of hydrolysis after ageing. The aged integrated polymer solution was cast into a watch glass and allowed to dry before being placed on a Perkin Elmer spectrum 100 FTIR-Attenuated total reflectance (ATR) spectrometer sensor for recording. The FTIR spectra were recorded at a spectrum resolution of 4 cm^−1^ with wave numbers ranging from 650–4000 cm^−1^ and an average of 32 scans. The absolute measurements provided by the ^1^H NMR analysis of the time zero samples were used as calibration points for the measurements of change in absorbance obtained from the FTIR in order to derive the absolute degree of hydrolysis of the aged polymer samples.

## 3 Results and disscussion

### 3.1 Degree of hydrolysis of integrated polymers in samples aged at high temperature

It is worth emphasizing that degree of hydrolysis is defined as the actual number of amide (CONH_2_) groups that could be replaced by carboxylate (COO^−^) groups [12]. Accordingly, in determining the level of hydrolysis of amide groups in the backbone of the water-soluble polymer structure, FTIR and NMR spectroscopy were utilised.

#### 3.1.1 FTIR analysis of degree of hydrolysis of PAM and PVP mix samples

The analysis in Table 3 shows FTIR spectrum assignment on overall PAM and PVP mix samples and the outcome indicates that the observed absorption peaks correspond to their microstructural functional group. Accordingly, the results indicate that the peaks at 3361-3298 cm^−1^ and 2984-2219 cm^−1^ correspond to primary amide (NH_2_) asymmetric and symmetric stretching respectively. Peaks at 2984-2219 cm^−1^ were assigned to secondary amide N–H stretching. Primary amide and secondary amide C=O stretching (CONH_2_) show prominent peaks at 1680-1630 cm^−1^ and 1629-1603 cm^−1^ respectively, and these peaks can be investigated to explore the interaction between PAM and PVP amide group hydrolysis. Furthermore, the peaks at 1496 -1492 cm^−1^ correspond to the asymmetric stretching of the C–N–C group, whereas the peak at 1492 -1420 cm^−1^ was assigned to the asymmetric stretching of the COO^−^ group. Then the peaks at 1320-1293 cm^−1^ were assigned to N–C stretching, while the peak at 1108-1098 cm^−1^ corresponds to C–O–C bending and those at 996-896 cm^−1^ were assigned to C–C symmetric-asymmetric stretching [21, 22, 23, 24].

**Table 3:** Assignment of FT-IR characterization bands ratio for overall PAM and PVP mix samples after ageing at 90 °C.

Peak Assignment	Overall PAM and PVP mix (wavenumber cm−1)
Primary amide NH_2_ asymmetric stretching	3361–3298
Secondary amide N–H stretching	2984–219
C–H Stretching	2159–2189
Primary Amide C=O Stretching (CONH_2_)	1650–1642
Secondary amide C =O Stretching (CONH_2_)	1639–1629
C–N–C Stretching	1496–1492
COO- Stretching	1492–1420
N–C Stretching	1320–1293
C – O–C Stretching	1108–1098
C–C symmetric - Asymmetric stretching	996–896

**Fig. 2:**
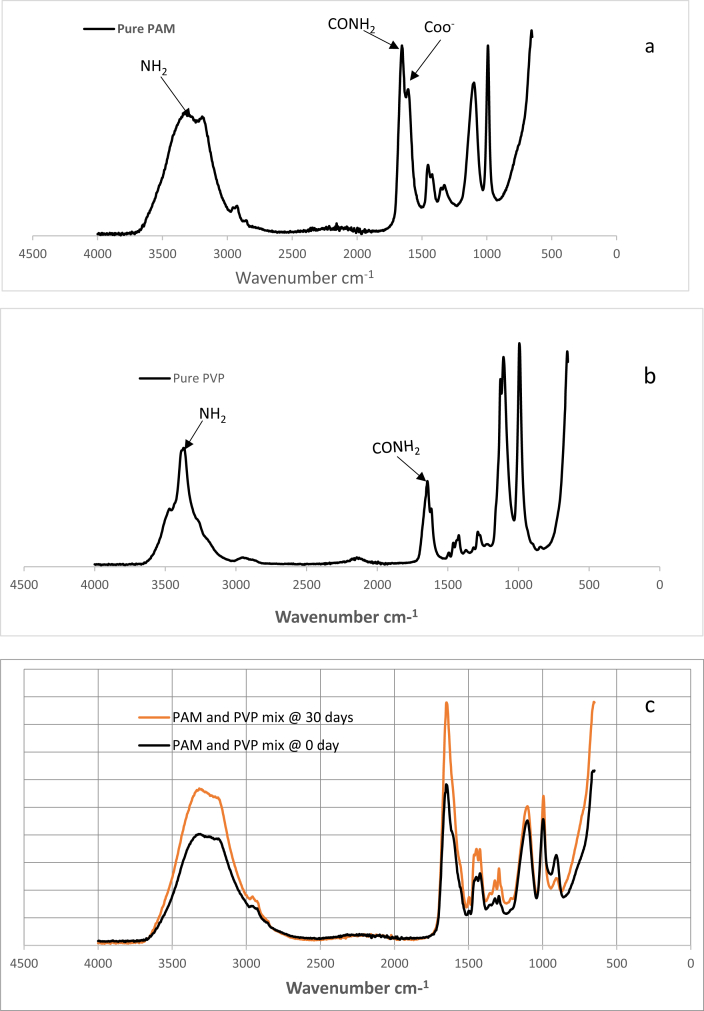
FT-IR absorbance spectra for (2a) pure PAM (2b) pure PVP and (2c) Comparative PAM and PVP mix samples at time 0 and 30 days after aging at 90 °C.

In line with Table 3, Fig. 2 (2a, 2b, 2c) presents the FT-IR absorbance spectra for pure PAM (2a) pure PVP (2b) and comparative plot of PAM and PVP mix samples at time 0 and 30 days (2c). The plot focuses on the absorbance change at C=O stretching of the CONH_2_ amide group because amide group (CONH_2_) hydrolysis was found to be the primary mechanism behind water soluble polymer (polyacrylamide) degradation [9]. However, at time zero, absorbance shows less intensities compared to the absorbance at 30 days.

#### 3.1.2 Determination of the stability of integrated polymer solutions

**Fig. 3:**
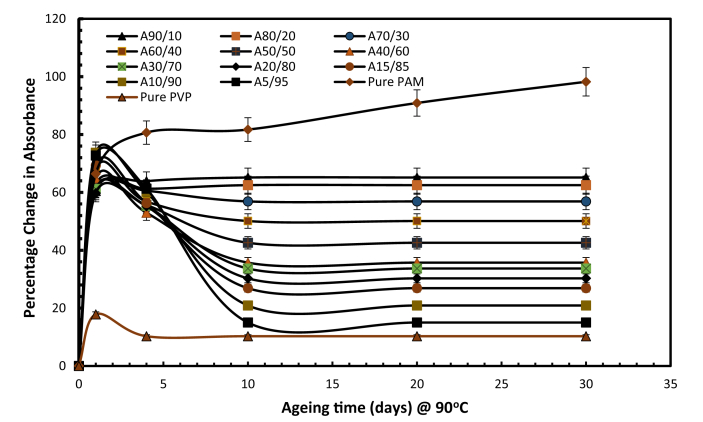
Percentage change in amide absorbance of pure PAM, pure PVP and various weight ratio of polymer mixtures in brine with TDS of 43,280 ppm at 90 °C (symbol A representing the PAM: PVP mixtures).

Fig. 3 indicates a plot evidence of percentage change in absorbance C=O stretching of the CONH_2_ amide group with prominent peak assigned at 1680 - 1603 cm^−1^, on integrating PAM and PVP against ageing time at 90 °C and a salinity of 43,280 ppm. The weight ratios with high proportions of PVP in the solution showed lower levels of amide absorbance, indicating less hydrolysis, whereas weight ratios with high PAM percentages exhibited high amide group absorbance, and thus more hydrolysis.

#### 3.1.3 NMR measurement of integrated polymer solutions at time zero

The ^1^H NMR analysis of integrated polymer using Bruker topspin 3.5 software shows a prominent peak positioned at 4.8 ppm, which represents the water content in the deuterium oxide solvent. Accordingly, the amounts of proton atoms (H) within the peak area were further calibrated. The calibrated point determine the amount of the proton atom (H) in the carbon skeleton with functional groups such as methine (CH), methylene (CH_2_) and methyl (CH_3_) or equivalent H atoms. This significantly, represent the molecular structure of the integrated polymer mix (PAM and PVP) being represented in Fig. 1 [21, 22].

**Fig. 4:**
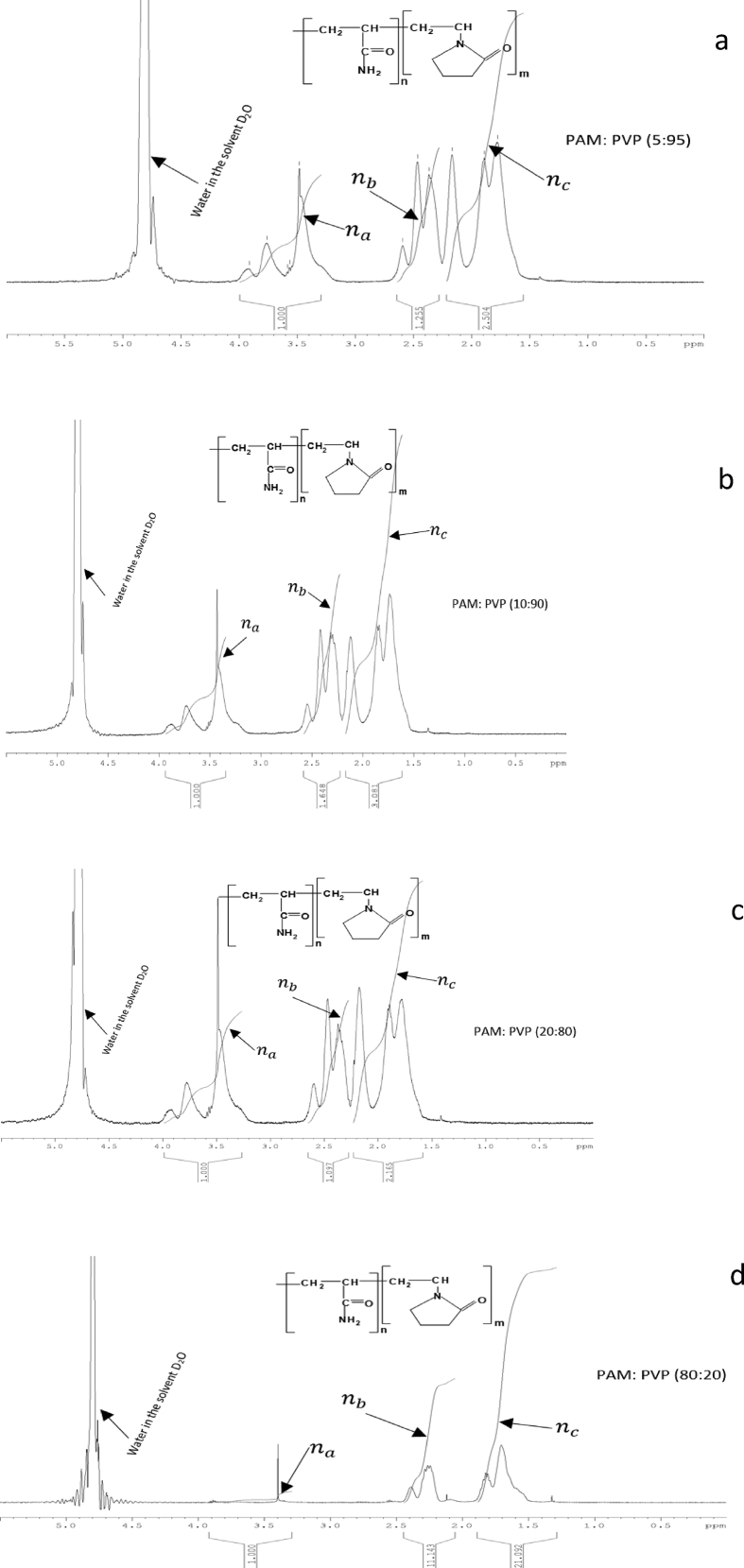
^1^H NMR spectra for the four selected weight ratio of polymer mixtures of PAM:PVP in brine with TDS of 43,280 ppm at 90 °C. In this figure, 4a is for A5/95 wt%, 4b is for A10/90 wt%, 4c is for A20/80 wt% and 4d for A80/20 wt% (symbol A representing the PAM: PVP mixtures). na, nb and nc present amide group, methylene amide group and methylene group, respectively.

^1^H NMR spectrum shown in Fig. 4 (4a, 4b, 4c and 4d) presents the spectra for the selected PAM: PVP polymer mixtures of A5/90 wt% (4a) A10/90 wt% (4b) A20/80 wt% (4c) and A80/20 wt% (4d) in brine with TDS of 43,280 ppm at 90 °C. From the figure, peaks at 3.4–3.8 ppm representing the proton (H) atom in functioning group of methine (CH) that is bonded to amide group in PVP as represented in $n_{a}$ whereas the peak at 2.00–2.5 ppm represent the methylene amide group (C=O) in PVP and the methine (CH) bonded to amide group (C=O) in PAM as represented in$\;n_{b}$. The peak at 1.50–1.90 ppm represents the methylene (CH_2_) group in PAM and PVP as represented in $n_{c}$[23, 24]. Accordingly, a three-step approach to the analysis of the degree of hydrolysis ($DH_{i})$ using proton NMR was adopted as shown in Eqs. (1a), (1b), (1c)

(1a) $$\text{Average}\;\text{peaks}\;\text{shift}\;\left(N_{\mathrm{avg}}\right)=\frac{1}{n}\underset{\text{i}=1}{\sum }N_{i}=\frac{1}{n}\;\left(N_{a}+N_{b}+N_{c}+\ldots \;N_{n}\right)$$

(1b) $$\text{Proton}\;\text{and}\;\text{Equivalent}\;\text{H}\;\text{atom}\;\text{total}\;\text{series}\;=\;Na+\frac{N_{b}}{N_{avg}}+\frac{N_{C}}{N_{avg}}$$

(1c) $$DH_{i}\text{(\%)}=\frac{\frac{N_{b}}{N_{avg}}}{Na+\frac{N_{b}}{N_{avg}}+\;\frac{N_{C}}{N_{avg}}\;}\;\times \;100$$

where, $N_{a}$ is the calibration point for methine (CH) proton in PVP, $N_{b}$ is methylene (CH_2_) proton bonded to amide group in PVP and the methine (CH) bonded to amide group (C=O) in PAM and $N_{c}$is the equivalent H atoms in the peak integration of the methylene (CH_2_) proton in PAM: PVP solutions.

The initial degrees of hydrolysis of the integrated polymers PAM:PVP dissolved in brine are recorded in Table 4. The results indicate that the initial degree of hydrolysis decreases with the increasing weight ratio of PVP in the integrated polymer solution.

**Table 4:** Initial degree of hydrolysis (*DH*_*i*_) for time zero ageing and brine sample with a salinity of 43,280 ppm at 90 °C.

Sample No:	Weight ratio composition (wt %)	DH_i_
1	A100/0	38%
2	A90/10	27%
3	A80/20	26%
4	A70/30	25%
5	A60/40	25%
6	A50/50	25%
7	A40/60	24%
8	A30/70	24%
9	A20/80	24%
10	A15/85	24%
11	A10/90	24%
12	A5/95	23%
13	A0/100	23%

For the initial samples at time zero, it is clear that the degree of hydrolysis rises when the weight proportion of PAM is higher than that of PVP. For instance, in weight proportion of A10/90 wt% the initial degree of hydrolysis was 27.4%, whereas for higher weight proportion of A90/10 wt % it was 44.5%. According to Uranta et al. [12], to convert the percentage absorbance change into degree of hydrolysis, the percentage increases in amide groups and initial degree of hydrolysis (DH) are combined in the final equation as expressed in Eq. (2).

(2) $$DH_{t}=\frac{100+\%CONH_{2}}{100}\times DH_{i}$$

where *DH*_*t*_ is the degree of hydrolysis at each designated time, *DH*_*i*_ is the initial degree of hydrolysis at time zero from the ^1^H NMR analysis, and %CONH_2_ is the percentage change in absorbance.

**Fig. 5:**
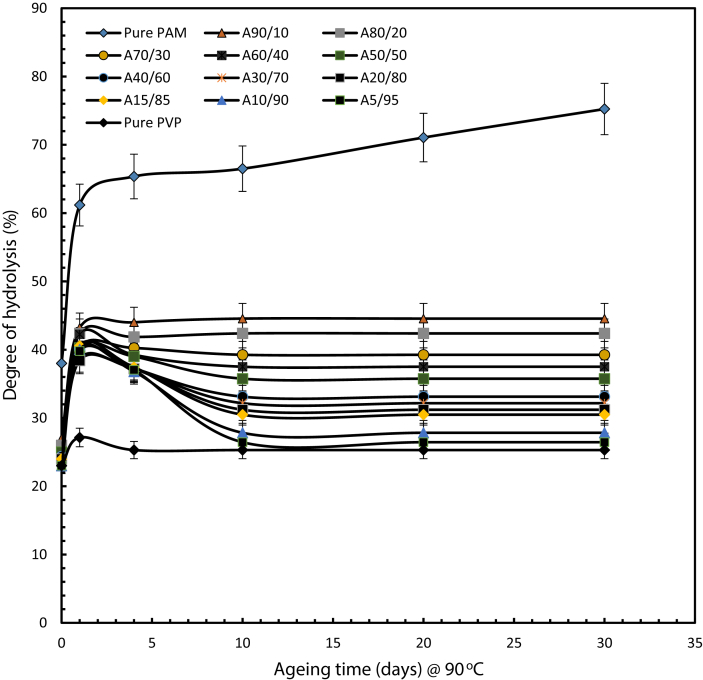
Calculated degree of hydrolysis for pure PAM, pure PVP and various weight ratio of polymer mixtures in brine with TDS of 43,280 ppm at 90 °C (symbol A representing the PAM: PVP mixtures).

Fig. 5 demonstrates values of the degree of hydrolysis of pure PAM and PVP, together with those of solutions of different weight ratios against ageing time up to 30 days in a brine of moderate salinity. The results show that a mixture solution with a high weight proportion of PVP exhibits less hydrolysis compared to pure PAM. The degree of hydrolysis of pure aged PAM after 30 days was reduced significantly from 75 % by adding 10 wt% of PVP and 90 wt % PAM, and the reduction continued linearly down to 44.55 %PAM. Further additions of PVP have a strong impact on PAM hydrolysis. This weight ratio is less susceptible to hydrolysis compared to 100% PAM. The degree of hydrolysis is constant for most mixtures from 4 days of ageing onwards and continuing up until 30 days.

### 3.2 Thermal stability of integrated PAM/PVP polymers at elevated temperature

**Fig. 6:**
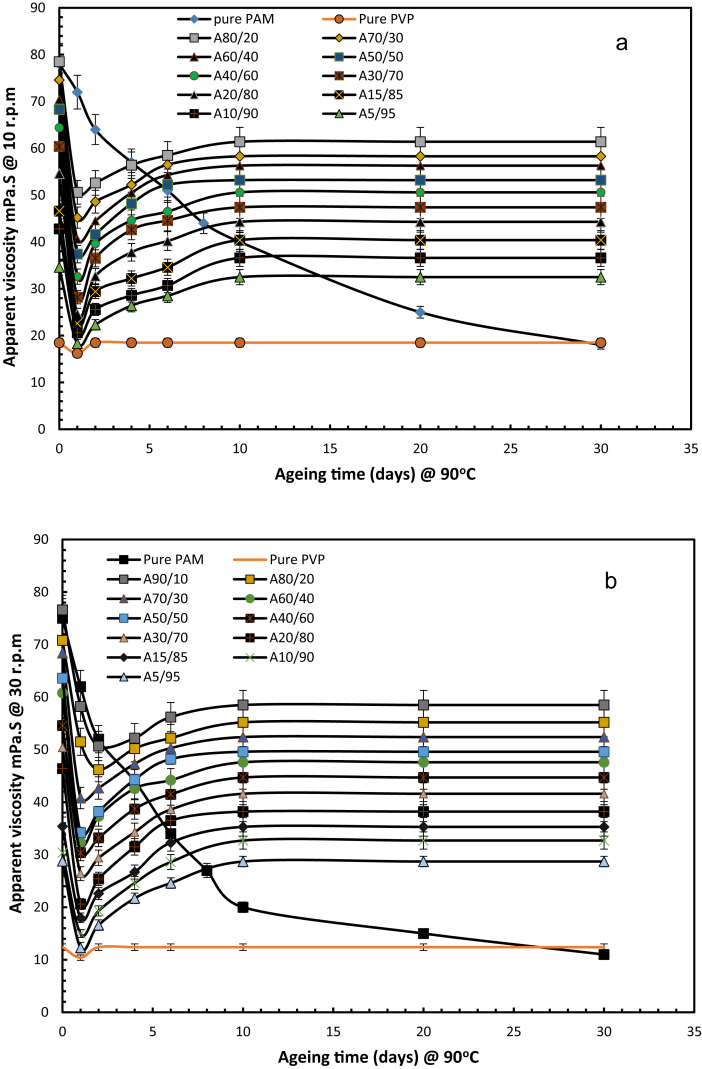
Viscosity of PAM and different weight ratios of integrated polymer at 90 °C and 43,280 ppm TDS at rotational speeds of (a) 10 rpm and (b) 30 rpm. (symbol A representing the PAM: PVP mixtures).

The viscosity of integrated polymer solutions for a range of weight ratios was measured at rotational speeds of 10 and 30 rpm, which correspond to shear rates of 17 and 51 s^-1^ the results are shown in Fig. 6 which indicates that the incorporation of PVP and PAM enhanced resistance to hydrolysis at temperature of 90 °C. Irrespective of rotational speed, the viscosity maintained stability from 10 days of ageing and continue till 30 days. That proved that PAM solution viscosity on addition of PVP showed high improvement in gel performance, when compare to 100 wt % PAM solution. This is because PVP is chemically inert and its addition to the copolymer led to resistance to degradation at high temperatures by protecting the PAM from extensive thermal hydrolysis, which would otherwise result in a loss of viscosity.

### 3.3 Optimization of the integrated polymer at elevated temperature and salinity

**Fig. 7:**
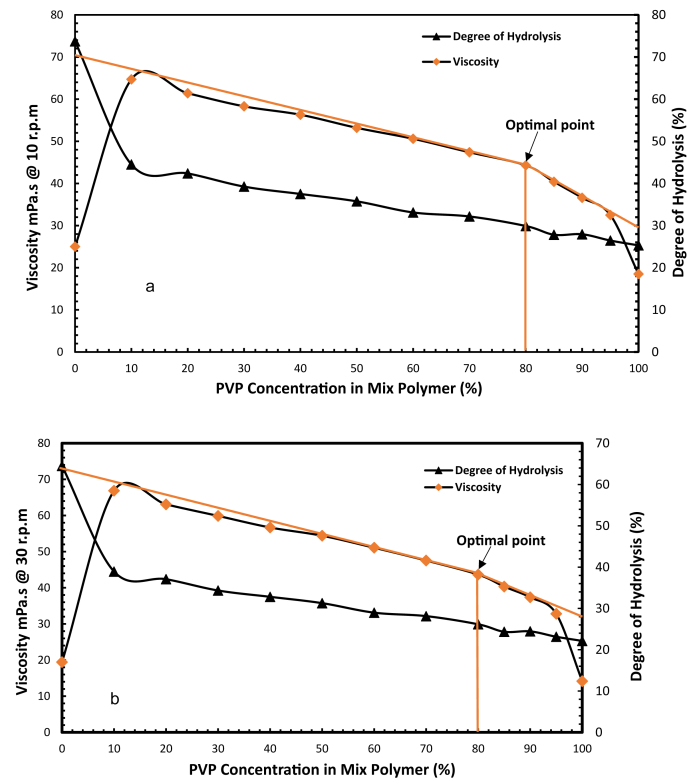
Determination of optimum concentration of PVP in integrated polymers of PAM: PVP at 90 °C and salinity of 43,280 ppm for rotational speeds of 10 rpm (a) and 30 rpm (b).

Fig. 7 shows plots of the viscosity of the integrated polymer solution and degree of hydrolysis against integrated polymer (PAM/PVP) concentration at rotational speeds of 10 and 30 rpm after 30 days. Both plots show similar trends. A rapid increase in viscosity can be observed from pure PAM to 10 wt% of PVP and then a linear drop in viscosity for solutions from 10 wt% of PVP to 80 wt% of PVP. After, 80 wt% of PVP concentration in mix polymers, the viscosity drop. The hydrolysis results mirror those seen for viscosity. A rapid decrease in degree of hydrolysis is seen from pure PAM to 10 wt% PVP followed by a much slower linear reduction for PVP concentrations greater than 10 wt%. The combination of 80 wt% of PVP and 20 wt% of PAM is considered to be the optimal point for effective application in reservoirs at a temperature 90 °C and formation water salinity of 43,280 ppm.

### 3.4 Effect of increased salinity on degree of hydrolysis and viscosity of optimal mixture of PAM and PVP

**Fig. 8:**
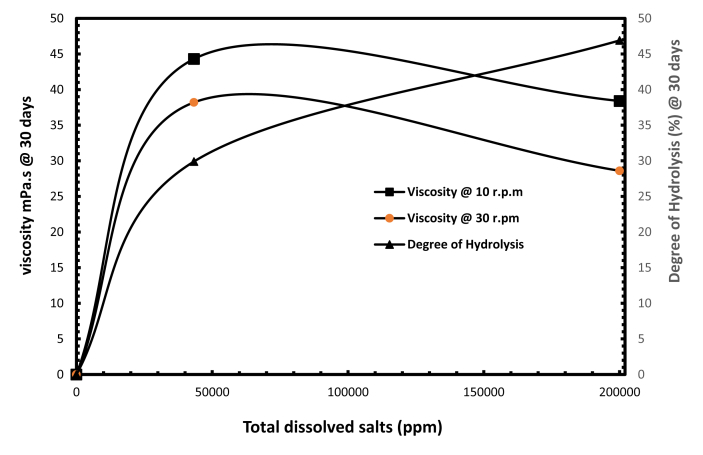
Impact of salinity concentration on optimised integration of PAM: PVP (20:80).

The incorporation of Polyvinylpyrrolidone (PVP) effectively protects polyacrylamide (PAM) against thermal hydrolysis in a solution of moderate salinity at 43,280 ppm TDS and a temperature of 90 °C. Further measurements were made to investigate the stability of the optimised mixture of PAM and PVP at the higher salinity of 200,000 ppm TDS. Fig. 8 shows plots of viscosity and degree of hydrolysis of the optimised integrated polymer solution A20/80 wt % against total dissolved salts (TDS) at 30 days. The figure shows that the degree of hydrolysis increases from 30 to 47% when the values of total dissolved salts increase from 43,280 ppm to 200,000 ppm. Meanwhile the viscosity drops sharply from 44 to 38.4 mPa.s respectively at 10 rpm. Similar trends are observed for the measurements at the higher shear rate of 30 rpm, and it is clear that an increase in total dissolved salts in the reservoir reduces gel stability by promoting a higher degree of hydrolysis.

### 3.5 Extending integrated polymer into extreme high salinity condition

In summary, a polymer weight ratio of A20/80 wt% has been found to be the optimal composition for a temperature of 90 °C and salinity of 43,280 ppm TDS. Furthermore the optimised composition point of A20/80 wt% needed to be assessed to see if it could withstand reservoir conditions of extremely high water salinity at 200, 000 ppm and a temperature of 90 °C. The results shown in Fig. 8 indicate an increase in degree of hydrolysis and reduced solution viscosity, leading to a loss of gel stability.

To ameliorate the effect of the challenging situation of extremely high salinity, 2-Acrylamido-2-MethylpropaneSulfonic acid (AMPS) was added to the optimised weight ratio of A20/80 wt %. The AMPS concentration in the optimised integrated polymer was screened at different weight percentages of 90, 50, 10, 5 and 0 wt%.

#### 3.5.1 Rate of hydrolysis of the amide group of integrated polymers in extreme salinity

FT-IR spectra were used to identify the absorbance of the amide group via peak assignment for the different weight ratios of PAM, PVP, and AMPS labelled as B in Table 5. In this table, the FT –IR spectrum indicating peak at 1638-1631 cm-1 could be assigned to the C=O stretching vibrations of CONH_2_. Apparently, the range between 3356 to 3349 cm^−1^ represent the primary amide NH_2_ and OH asymmetric stretching vibrations of CONH_2_, while the peaks at 1463-1553 cm^−1^ are assigned to CH_3_ and CH_2_ group stretching. Those in the range 1294–1296 cm^−1^ are assigned to C–N–C stretching, and those between 1115 -1188 cm^−1^ to C–N stretching. Tables 6 and 7 show the results of peak assignment for optimised polymer mixture of A20/80 wt% and pure AMPS in a brine with 200,000 ppm salinity, respectively. As can be seen from these tables, the AMPS structure with the bands for the sulfonate (SO_3_) functional groups and their stretching appearing in the range 1043–1044 cm-1 [25, 26, 27, 28], whereas those for C–C symmetric asymmetric stretching are at 995-996 cm^−1^.

**Table 5:** FTIR spectra or peak assignment for various weight ratio of polymer mixtures (symbol B representing PAM: PVP: AMPS mixtures).

Peak Assignment	weight ratio composition (wavenumber cm^−1^)			
	B19/76/5	B18/72/10	B10/40/50	B2/8/19
Primary amide NH_2,_ and OH asymmetric stretching	3356	3333–3335	3349	3344–3354
Secondary amide N–H stretching	2022–2185	2038–2067	2039–2169	2032–2089
Primary Amide C=O Stretching	1638–1645	1632–1638	1637–1638	1631–1644
CH_3_ and CH_2_ stretching	1466–1467	1465–1466	1551–1553	1463–1467
C–N–C Stretching	1296	1295	1296	1294
C–N Stretching	1116	1115–1119	1187–1188	1118–1188
SO_3_ Stretching	1044–1043	1044–1043	1044–1043	1044–1043
C–C symmetric - Asymmetric stretching	996–997	991–996	995–997	996–997

**Table 6:** FTIR peak assignment for optimised polymer mixture of A20/80 wt % in a brine with 200,000 ppm TDS.

A20/80 wt % @ 200000 ppm TDS	
Peak Assignment	A80/20 wt % (wavenumber cm^−1^)
Primary amide NH_2_ asymmetric stretching	3345–3333
Secondary amide N–H stretching	2162–2134
C–H Stretching	2096–2053
Primary Amide C=O Stretching	1642–1634
Secondary amide C =O Stretching	1633–1609
C–N–C Stretching	1493–1457
COO- Stretching	1450–1449
N–C Stretching	1296–1295
C – O–C Stretching	1117–1113
C–C symmetric - Asymmetric stretching	997–901

**Table 7:** FTIR peak assignment for pure AMPS in a brine with 200,000 ppm TDS.

Pure AMPS @ 200000 ppm TDS	
Peak Assignment	Pure AMPS (wavenumber cm^−1^)
Primary amide NH_2,_ and OH asymmetric stretching	3358–3347
Secondary amide N–H stretching	2162
Primary Amide C=O Stretching	1636–1638
CH_3_ and CH_2_ stretching	1551–1553
C–N–C Stretching	1296
C–N Stretching	1187–1189
SO_3_ Stretching	1044
C–C symmetric - Asymmetric stretching	995–997

**Figure 9:**
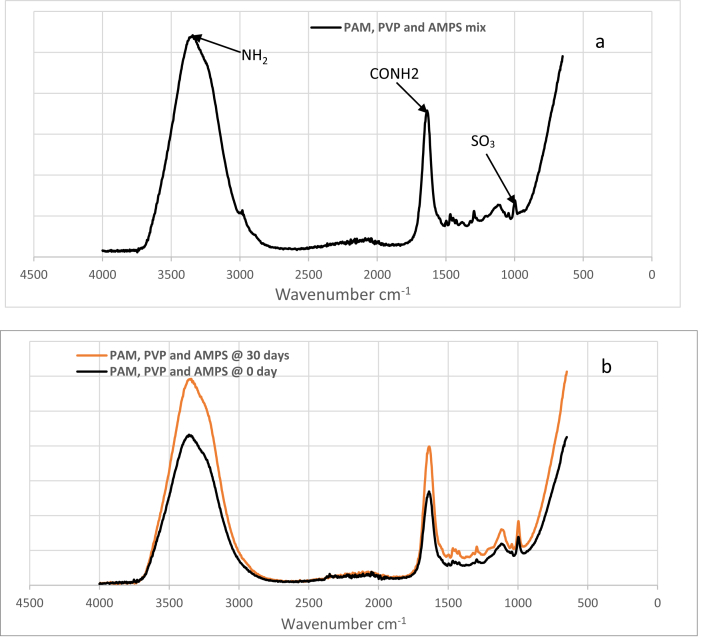
FT-IR absorbance spectra for (9a) PAM, PVP and AMPS (9b) Comparative PAM, PVP and AMPS mix samples at time 0 and 30 days.

**Fig. 10:**
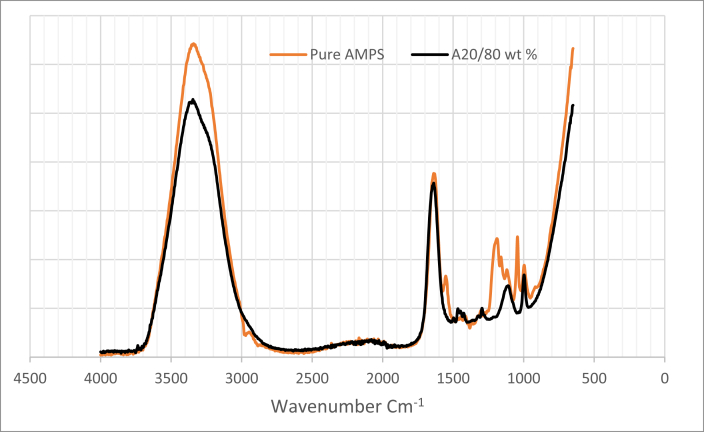
FT-IR spectrum for pure AMPS and optimised polymer composition of A20/80 wt % in brine of 200,000 ppm TDS and temperature of 90 °C. (symbol A representing the PAM: PVP mixtures).

**Fig. 11:**
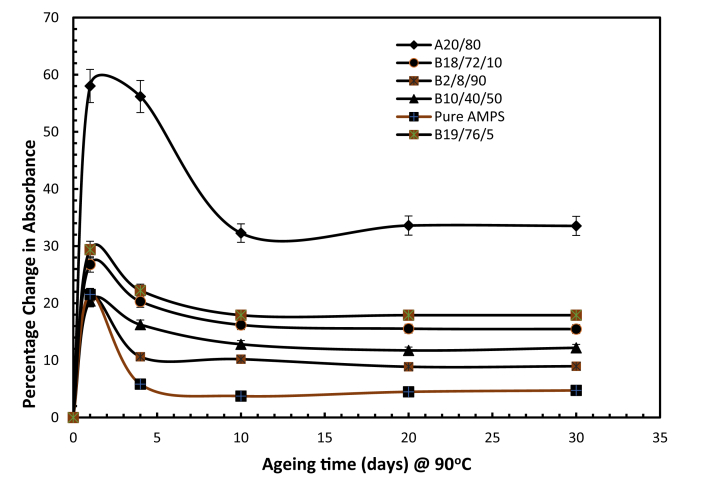
Percentage change in amide absorbance polymer weight ratio composition at 90 °C and 200,000 ppm TDS. (symbol A and B representing the PAM: PVP mixture and the PAM:PVP:AMPS mixtures, respectively).

The FT-IR spectra for PAM, PVP and AMPS mix and comparative PAM, PVP and AMPS mix samples at time 0 and 30 days are shown in Fig. 9. Additionally, Fig. 9 presents a comparative plot of PAM, PVP and AMPS mix samples at time 0 and 30 days respectively where at time zero, absorbance shows less intensities compared to the absorbance at 30 days. Accordingly, from Fig. 10, the FT-IR Spectrum for pure AMPS and optimised polymer composition of A20/80 wt% in brine of 200,000 ppm TDS and temperature of 90 °C. It can be seen in this figure that pure AMPS exhibited higher absorbance peak compared to the optimised A20/80 wt%. The summary of calculated percentage change in absorbance for integrated polymers at 90 °C in a brine with 200,000 ppm salinity were given in Fig. 11.

As can be seen in Fig. 11, high percentages of amide absorbance were observed of up to 58 and 56% respectively after the second and fourth days of ageing; however, the levels declined gradually with ageing until 10 days and subsequently remained stable. The integrated mixture of PAM: PVP with 90% by weight of AMPS reduced amide absorbance significantly from 30% down to 9 % after 30 days of ageing.

**Fig. 12:**
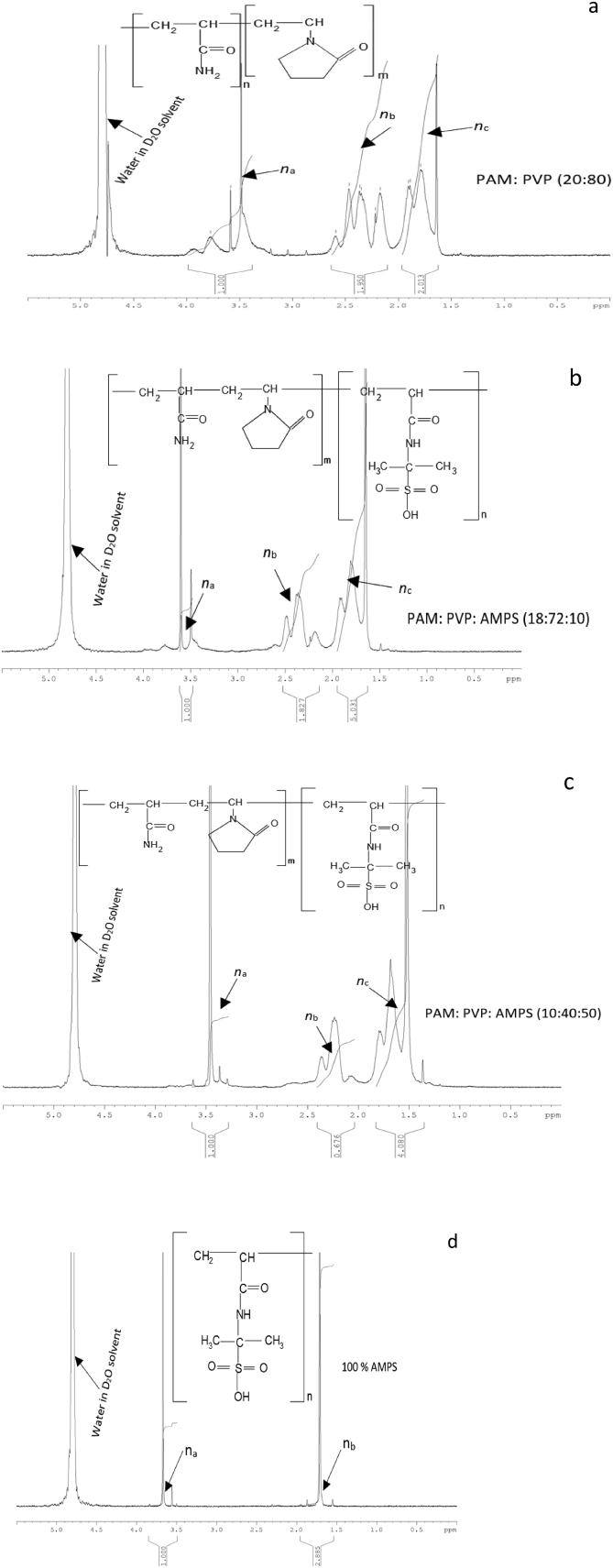
^1^H NMR spectra for the four selected weight ratio of polymer mixtures of PAM:PVP and PAM:PVP:AMPS in brine with TDS of 200,000 ppm at 90 °C. In this figure, 12a is for A 20/80 wt%, 12b is for B18/72/10 wt%, 12c is for B10/40/50 wt% and 12d is for 100% AMPS (symbol A and B representing the PAM: PVP mixture and the PAM:PVP:AMPS mixtures, respectively). na, nb and nc present amide group, methylene amide group and methylene group, respectively.

Fig. 12 presents the ^1^H NMR spectra for the integrated weight ratio of PAM: PVP polymers 20:80 wt % (12a) and spectra for integrated mixtures of PAM: PVP: AMPS polymers of 18/72/10 wt% (12b), 10/40/50 wt% (12c) and pure AMPS (12d) at temperature of 90 °C and salinity of 200000 ppm. The peak at 4.8 ppm represents water content in the deuterium oxide (D_2_O) solvent. However, the peak observed between 3.4 - 3.2 ppm could be attributed to the equivalent hydrogen (H) atom of the CH group bonded to SO_3_ in AMPS and the amide group (C=O) in the PVP structure [25]. The peaks between 2.20 - 2.40 ppm and 1.60–1.90 ppm are assigned to the equivalent hydrogen (H) atom of the CH group bonded to the amide group (CONH_2_). The peaks measured at 1.60–1.90 ppm represent the equivalent atoms of the CH_2_ group in PAM, PVP and AMPS respectively. In this set of results, n_a_ is defined as equivalent H atom in peak integration for CH group for PVP and AMPS bonded to amide group (C=O), whereas n_b_ is the equivalent H atoms in peak integration for CH group bonded to amide group (C=O) combined PAM and n_c_ equivalent H atoms in peak integration for of CH_2_ group for PAM and PVP.

The initial degrees of hydrolysis for integrated polymer solutions were evaluated using Buker Topsin 3.5 software and the results are presented in Table 8. It can be seen that a small amount of AMPS (5 wt%) gives a 15% reduction in degree of hydrolysis Further additions of AMPS up to 90 wt% see only a 2% further reduction of hydrolysis, which implies that no extra benefit is gained by adding more than 5 % of AMPS to the mixture.

**Table 8:** Initial degree of hydrolysis (*DH*_*i*_) for brine samples of 200,000 ppm salinity at 90 °C.

Sample No:	PAM wt%	PVP wt%	AMPS wt%	DH_i_
1	20	80	0	35
2	19	76	5	20
3	18	72	10	19
4	10	40	50	18
5	2	8	90	18

**Fig. 13:**
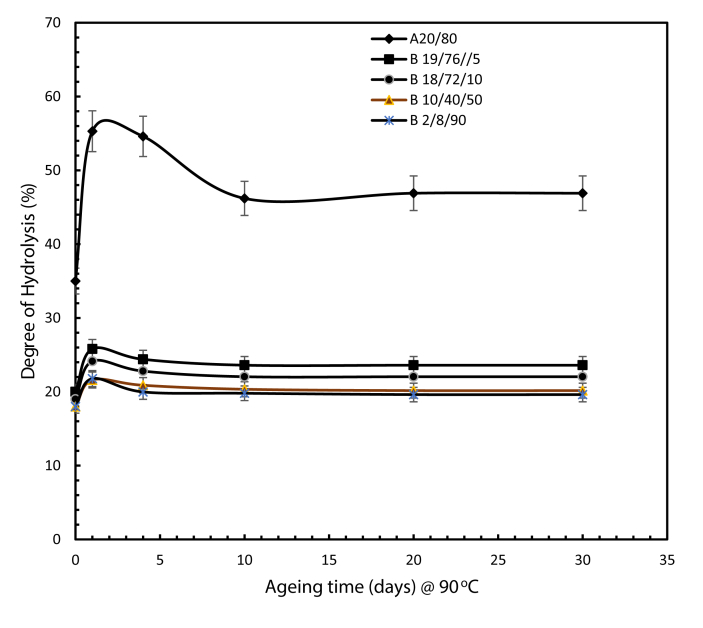
Extent of degree of hydrolysis of polymers of different weight ratio compositions at 90 °C and 200,000 ppm salinity. (symbol A and B representing the PAM: PVP mixture and the PAM:PVP:AMPS mixtures, respectively).

In determining the degree of hydrolysis at the designated ageing times, the percentage absorbance is converted into degree of hydrolysis using the same procedure that was adopted for the PAM: PVP mixtures. Fig. 13 shows the degree of hydrolysis of integrated polymer solutions in high temperature and high salinity conditions as a function of time. The integrated PAM: PVP: AMPS polymers exhibit lower degrees of hydrolysis of about 22% compared to 46% for the PAM: PVP solutions.

### 3.6 Stability of integrated PAM: PVP: AMPS polymers in extremely high salinity conditions

**Fig. 14:**
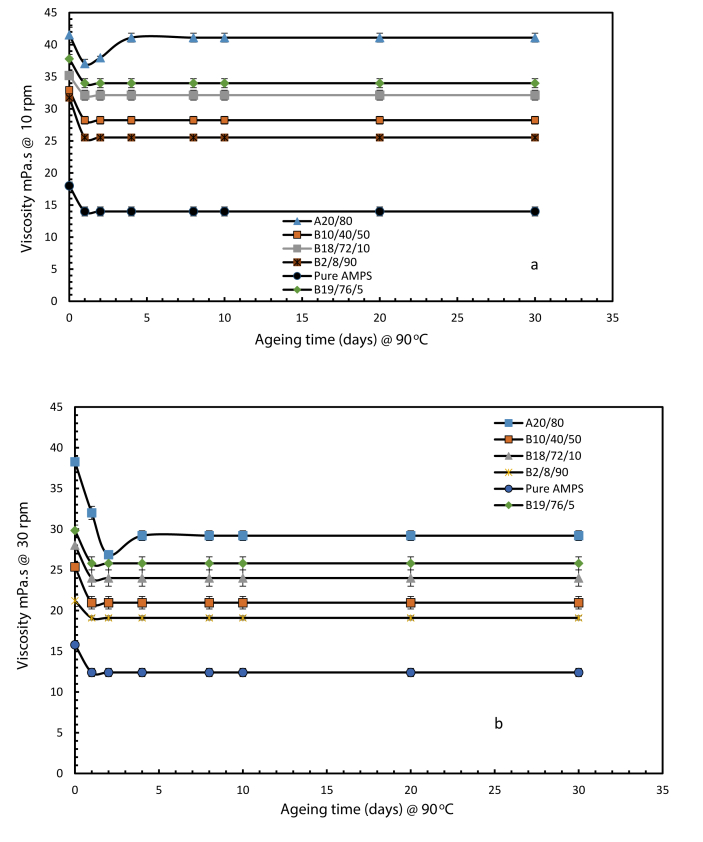
Viscosity of PAM: PVP: AMPS polymers of different weight ratio compositions at 90 °C in salinity of 200,000 ppm TDS at (a) 10 rpm and (b) 30 rpm. (symbol A and B representing the PAM: PVP mixture and the PAM:PVP:AMPS mixtures, respectively).

Fig. 14 illustrates changes in viscosity against ageing time for two rotational speeds of 30 and 10 rpm. The figure demonstrates that the incorporation of AMPS in the optimised PVP and PAM mixture exhibited good stability in high salinity conditions of 200,000 ppm TDS and temperature of 90 °C. The evidence showcase that the viscosity stabilised beginning from 2 days of ageing and continue till 30 days whereas for the A20/80 wt % in 200, 000 ppm TDS and temperature of 90 °C the viscosity stabilised from 4 days of ageing and continue till 30 days.

### 3.7 Optimization of integrated polymer solutions in extremely high salinity

The optimization of the weight ratio composition of the PAM: PVP: AMPS polymer mixture will provide more effective oilfield operations in enhanced oil recovery, especially in terms of the economic evaluation and feasibility of the process.

**Fig. 15:**
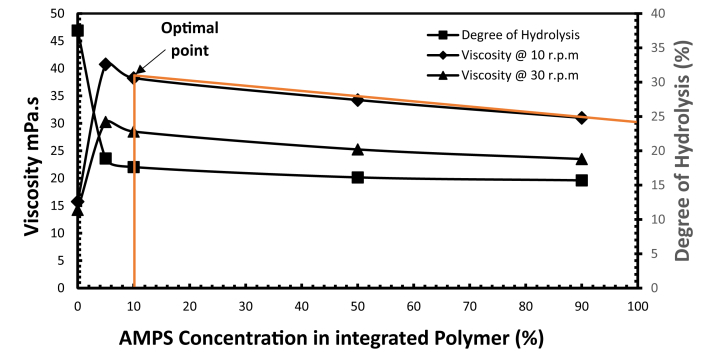
Optimised weight proportion of PAM: PVP: AMPS at 90 °C and 200,000 ppm TDS.

Fig. 15 demonstrates how the optimised concentration of AMPS in the integrated polymer mixture was selected according to the results over 30 days of ageing. However, the weight proportion of 10 wt % AMPS was found to be optimal, resulting in the overall optimum composition of the PAM:PVP:AMPS at weight percentages of B18/72/10 wt % for use at a temperature of 90 °C and salinity of 200,000 ppm.

In summary, Gao et al. [19] reported on the specifications used for the application of hydrolysed PAM based on experience at the Shengli Company. They stated that hydrolysed PAM product should go through screening tests before being deployed in the field and that two criteria should be met: the degree of hydrolysis should be below 25%; and the hydrolysed solution must maintain good stability and viscosity greater than 11.5 mPa.s against the influence of shearing, temperature and water salinity. However, the results in Fig. 14 show that the polymer mixture proposed in the present work has surpassed those criteria, achieving a degree of hydrolysis of 22.04% with good stability and viscosity maintained at 30.6 mPa.s at the low shearing rate of 10 rpm (17 s^-1^) in the presence of extremely high salinity at 200,000 ppm and at a temperature of 90 °C.

## 4 Conclusions

The challenging reservoir conditions of high temperature and high salinity drove this work to improve the performance of polymers and optimizing the conditions using polymer integration technique (PIT). In view of that, the followings conclusions were drawn:

(1) The incorporation of Polyvinylpyrrolidone (PVP) effectively protects polyacrylamide (PAM) against thermal hydrolysis by drastically reducing the degree of hydrolysis of pure PAM from 75% to 29.9% with associated increases in viscosity from 11 to 38.2 mPa.s and from 18 to 44.3 mPa.s corresponding to rotational speeds of 30 and 10 r.p.m respectively at 30 days of sample ageing. Giving an optimizing fit of PVP weight proportion of 80 wt % as the optimum concentration, leading to overall optimum composition of A20/80 wt % for use at a temperature of 90 °C and in moderate salinity of 43,280 ppm.
(2) Adding AMPS to the PAM:PVP mixture can help to stabilise the copolymer gel at higher salinity. 10 wt % of AMPS was found to be the optimal concentration, giving an overall optimum ter-polymer composition of B18/72/10 wt % PAM:PVP:AMPS for use at a temperature of 90 °C and in a salinity of 200,000 ppm.
(3) The incorporation of the AMPS into the optimised mixture of A20/80 wt % boosted the capacity of the polymer solution by decreasing the degree of hydrolysis from 29.9 to 22.04%.

## Declarations

### Author contribution statement

Kingsley Uranta: Performed the experiments; Analyzed and interpreted the data; Wrote the paper.

Sina Rezaei Gomari: Conceived and designed the experiments; Analyzed and interpreted the data; Contributed reagents, materials, analysis tools or data; Wrote the paper.

Paul Russell, Faik Hamad: Conceived and designed the experiments; Analyzed and interpreted the data; Wrote the paper.

### Funding statement

This work was supported by the Petroleum Technology Development Fund (PTDF) in Nigeria.

### Competing interest statement

The authors declare no conflict of interest.

### Additional information

No additional information is available for this paper.
